# Moringin Pretreatment Inhibits the Expression of Genes Involved in Mitophagy in the Stem Cell of the Human Periodontal Ligament

**DOI:** 10.3390/molecules24183217

**Published:** 2019-09-04

**Authors:** Luigi Chiricosta, Agnese Gugliandolo, Francesca Diomede, Jacopo Pizzicannella, Oriana Trubiani, Renato Iori, Giuseppe Tardiolo, Simone Guarnieri, Placido Bramanti, Emanuela Mazzon

**Affiliations:** 1 IRCCS Centro Neurolesi “Bonino-Pulejo”, Via Provinciale Palermo, Contrada Casazza, 98124 Messina, Italy (L.C.) (A.G.) (G.T.) (P.B.); 2 Department of Medical, Oral and Biotechnological Sciences, University “G. d’Annunzio” Chieti-Pescara, 66100 Chieti, Italy (F.D.) (J.P.) (O.T.); 3 Consiglio per la ricerca in agricoltura e l’analisi dell’economia agraria, Centro di ricerca Agricoltura e Ambiente (CREA-AA), Via di Corticella 133, 40128 Bologna, Italy; 4 Department of Neuroscience, Imaging and Clinical Sciences, University “G. d’Annunzio”, Chieti-Pescara, 66100 Chieti, Italy; 5 Center on Aging Science and Translational Medicine (Ce.S.I.-Me.T.), University “G. d’Annunzio”, Chieti-Pescara, 66100 Chieti, Italy

**Keywords:** moringin, mitochondria, mitophagy, apoptosis, human periodontal ligament stem cells, next generation sequencing, neurodegenerative disorders

## Abstract

Moringin [4-(α-L-rhamnosyloxy) benzyl isothiocyanate] is an isothiocyanate extracted from *Moringa oleifera* seeds. It is an antioxidant known for several biological properties useful in the treatment of neurodegenerative diseases. Several neurodegenerative disorders such as Parkinson’s and Alzheimer’s diseases are linked to dysfunctional mitochondria due to the resulting increase of Reactive Oxygen Species (ROS). Stem cell-based therapeutic treatments in neurodegenerative diseases provide an alternative strategy aimed to replace the impaired tissue. In this study were investigated the deregulated genes involved in mitophagy in the human periodontal ligament stem cells pretreated with moringin. The RNA-seq study reveals the downregulation of *PINK1,* with a fold change (FC) of −0.56, such as the genes involved in the phagophore formation (*MAP1LC3B* FC: −0.73, *GABARAP* FC: −0.52, *GABARAPL1* FC: −0.70, *GABARAPL2* FC: −0.39). The moringin pretreatment downregulates the pro−apoptotic gene *BAX* (−0.66) and upregulates the anti-apoptotic genes *BCL2L12* (FC: 1.35) and *MCL1* (FC: 0.36). The downregulation of the most of the caspases (*CASP1* FC: −1.43, *CASP4* FC: −0.18, *CASP6* FC: −1.34, *CASP7* FC: −0.46, *CASP8* FC: −0.65) implies the inactivation of the apoptotic process. Our results suggest that mitochondrial dysfunctions induced by oxidative stress can be inhibited by moringin pretreatment in human periodontal ligament stem cells (hPDLSCs).

## 1. Introduction

**Figure 1:**
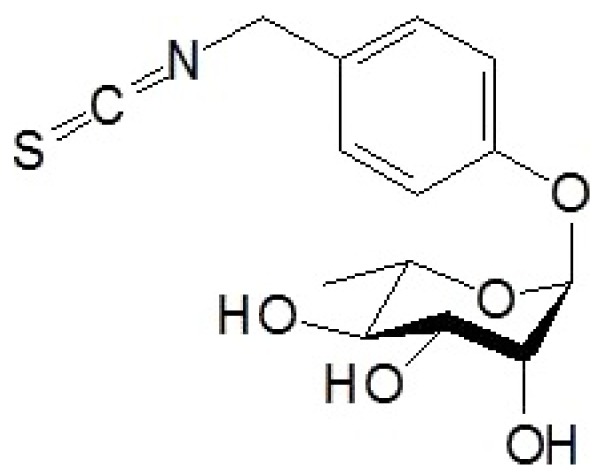
Chemical structure representation of the bioactive compound moringin extracted from *Moringa oleifera*.

Isothiocyanates are a class of phytocompounds obtained by hydrolysis of glucosinolates. Isothiocyanates have generated great interest in the clinical field for their pharmacological properties, ranging from cancer prevention [1] to neuroprotective effects [2]. Moringin (Figure 1) is an isothiocyanate extracted from *Moringa olei*fera, also called “miracle tree” [3]. 

In particular, moringin is the bioactivate form of glucomoringin and it is renowned to promote healthy benefits at doses of 0.1–1 g/kg due to its anti-inflammatory, anti-fibrotic, antimicrobial, anti-hyperglycemic, antioxidant and anti-tumour properties [4]. Additionally, moringin mitigates the neurodegeneration process and promotes neuroprotective effects through the suppression of proinflammatory mediators, protection from oxidative damage and the reduction of apoptosis [5,6]. Our research group has already studied the effect of moringin treatment on stem cells [7]. In particular, human Periodontal Ligament Stem Cells (hPDLSCs) pretreated with moringin differentiate into neuronal cells and, consequently, they could support neurological therapies [8]. Indeed, mesenchymal stem cells are immunoprivileged cells useful in regenerative medicine [9]. hPDLSCs have a fibroblastic-like morphological shape and are characterized by several mesenchymal and stemness markers [10]. They can spontaneously diversify into osteogenic, chondrogenic and adipogenic lineages. Because of their origin from the neural crest, hPDLSCs have the innate potential to differentiate into neuronal cells and, consequently, they are good candidates in neurodegenerative disease studies [11,12,13,14,15,16]. Moreover, they can be easily isolated from the oral cavity and represent an ideal model of mesenchymal stem cells. Our group has already shown that hPDLSCs have a neuroprotective effect against autoimmune encephalomyelitis, an experimental model of multiple sclerosis [17]. The ethiopathogenesis of the neurodegenerative diseases seems to correlate with mitochondria dynamics, morphology, motility, activity and oxidative stress [18,19,20]. Mitochondrial homeostasis is mediated by fission and fusion processes [21]. In particular, fission is required to generate novel mitochondria and contribute to quality control, while fusion reduces cellular stress mixing the impaired mitochondria. The elimination of the damaged mitochondria is mediated by mitophagy, a selective type of autophagy that has recently drawn the scientific interest [22]. In mammalian cells, mitophagy is induced by different pathways such as the PINK1/Parkin-mediated pathway and Outer Mitochondrial Membrane (OMM) receptor-mediated pathway [23]. The PINK1/Parkin-mediated is the best characterized and PINK1 protein plays the pivotal role in this process [24]. Under normal conditions, PINK1 acts as stress sensor involved in the detection of mitochondrial quality. Changes in the expression of PINK1 appear related to neurodegenerative disorders such as Alzheimer’s and Parkinson’s diseases [23,25]. In addition, several genetic mutations of PINK1 and Parkin are implicated [26,27]. These mutations generate an accumulation of impaired mitochondria and aggregation of proteins that lead to neuronal decline [28]. The purpose of this study was the investigation of the mitophagic and apoptotic processes in hPDLSCs pretreated with moringin (hPDLSCs-MOR). In particular, the RNA-seq analysis was performed to evaluate changes in the expression profile of the hPDLSCs-MOR compared to untreated hPDLSCs (hPDLSCs-CTR).

## 2. Results

### 2.1. Morphological Analysis and Proliferation Rate of hPDLSCs-MOR and hPDLSCs-CTR

**Figure 2:**
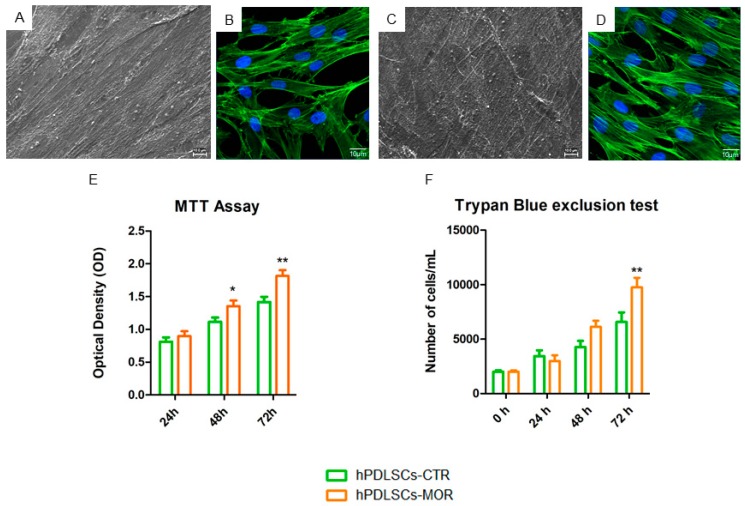
SEM analysis and evaluation rate of hPDLSCs-MOR and hPDLSCs-CTR proliferation. The SEM analysis is shown for the untreated (**A**) and treated (**C**) cells. The immunofluorescence for actin confirmed that the morphology of hPDLSCs-MOR (**D**) was not modified compared to hPDLSCs-CTR (**B**). Green fluorescence: cytoskeleton actin. Blue fluorescence: cell nuclei. The proliferation rate of hPDLSCs-MOR results higher when compared to the hPDLSCs-CTR (**E**). MTT analysis is then confirmed by Trypan Blue exclusion test (F). * *p* < 0.05; ** *p* < 0.01.

hPDLSCs were treated with moringin at 0.5 µM. The in vitro biological characteristics of hPDLSCs-CTR and hPDLSCs-MOR were evaluated using Scanning Electron Microscopy (SEM) images. The cells were cultured and expanded on a plastic dish and they exhibited a similar fibroblast-like morphology and plastic-adherence on the substrate. Moringin treatment did not modify the cell morphology visible under SEM observation (Figure 2A,C) and immunofluorescence for actin (Figure 2B,D). The growth curves of untreated and treated hPDLSCs showed a logarithmic trend. The moringin treatment increases the hPDLSCs proliferation rate in all considered time points, in particular the main effect was detected at 72 h (Figure 2E). Trypan blue exclusion test confirmed the MTT assay data (Figure 2F). 

### 2.2. Transcriptome Investigation of hPDLSCs-MOR and hPDLSCs-CTR

The transcriptome of hPDLSCs that received moringin treatment (0.5 µM for 48 h) and hPDLSCs that did not receive any treatment were explored by Next Generation Sequencing (NGS) analysis. An in-deep analysis of the global gene expression displayed that 4390 statistically relevant genes. Among them, 12 genes are exclusively expressed in hPDLSCs-MOR whereas 10 are exclusively included in the hPDLSCs-CTR. The remaining 4364 genes are in common between the two groups (Table S1). 

### 2.3. Genes Pathway Distribution

**Figure 3:**
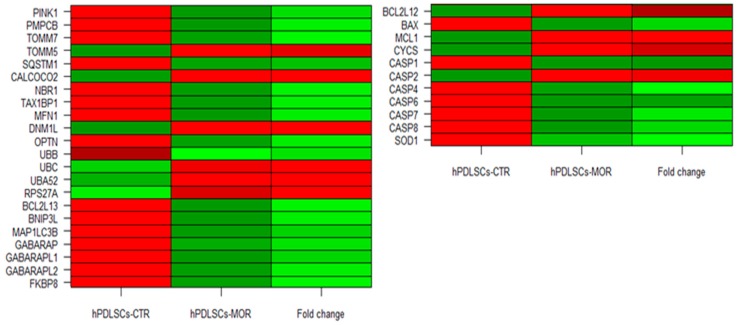
Heatmap of genes included in the analysis. In *hPDLSCs-CTR* and in *hPDLSCs-MOR* the green color represents a lower expression while the red color a higher expression. In *fold change*, the green color represents a downregulation in hPDLSCs-MOR while red represents an upregulation.

**Figure 4:**
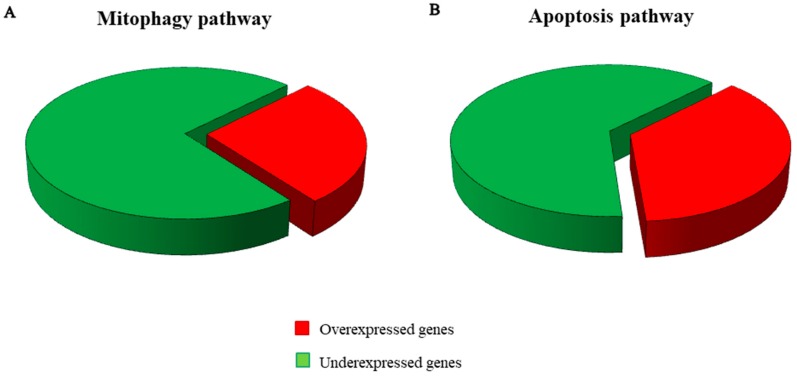
Distribution of genes involved in the mitophagy pathway (6 genes are overexpressed, 16 are underexpressed) (**A**) and in the apoptosis pathway (4 genes are overexpressed, 7 are underexpressed) (**B**).

**Figure 5:**
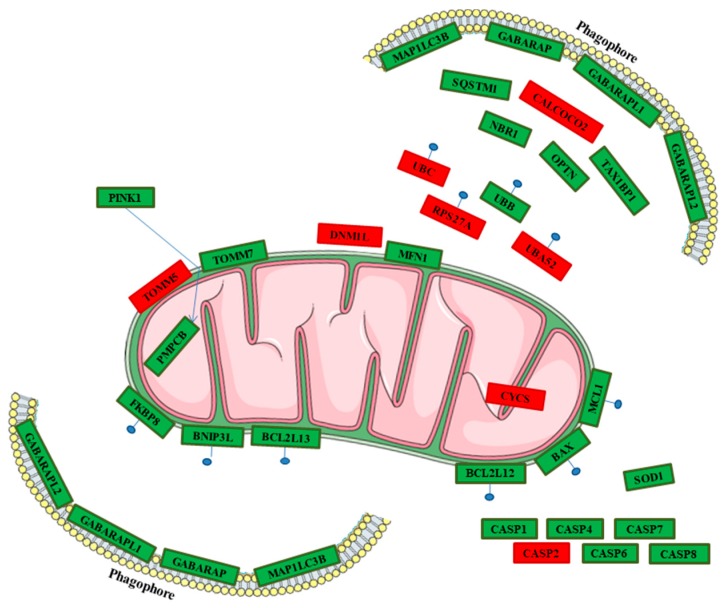
Genes expressed both in hPDLSCs-CTR and hPDLSCs-MOR involved in mitophagy and apoptotic pathways. All the represented genes are deregulated in hPDLSCs-MOR compared to hPDLSCs-CTR. In green are highlighted the genes found downregulated in hPDLSCs-MOR, whereas in red the genes found upregulated in hPDLSCs-MOR. Figure drawn using the vector image bank of Servier Medical Art by Servier (http://smart.servier.com/). Licensed under a Creative Commons Attribution 3.0 Unported License (https://creativecommons.org/ licenses/ by/3.0/).

**Figure 6:**
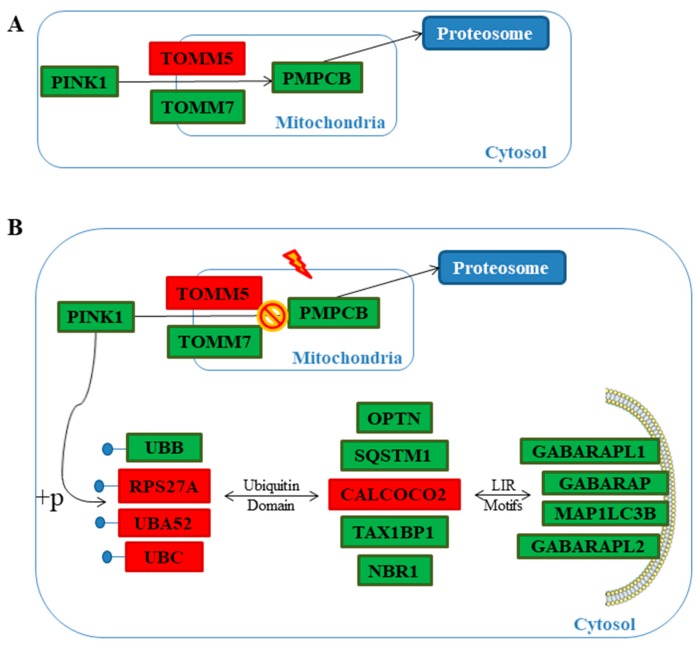
Genes expressed both in hPDLSCs-CTR and hPDLSCs-MOR involved in mitophagy pathway. In green are highlighted the genes found downregulated in hPDLSCs-MOR, whereas in red the genes found upregulated in hPDLSCs-MOR. Under physiological condition PINK1 is recruited in mitochondria through the TOM complex, is degraded by PMPCB and finally degraded by the protosome (**A**). Under stress conditions, PINK1 is retained in complex with TOM promoting the phosphorylation of ubiquitin; autophagy receptors are characterized both by P-UB domain and LIR motifs and they finally activate autophagy machinery (**B**).

The Reactome database collects a set of genes interaction pathways. It was used to analyse the genes differently expressed in our transcriptome. The investigation was focused on the 4364 genes in common between the transcription profiles of the hPDLSCs-CTR and hPDLSCs-MOR whereby the section “Analyze data”. Reactome was able to recognize 2906 genes. In particular, it was investigated the “mitophagy” pathway. To achieve the state-of-the-art, genes were integrated from the reviews of the last five years using the keywords “mitophagy” and “pink1 mediated” or “parkin independent”, “apoptosis” and “mitochondrial dynamics” and “neurodegeneration”. In Table 1 are listed the 22 genes related to mitophagy. In Table 2 are listed the 11 genes linked to apoptosis and oxidative stress. Figure 3 displays a heatmap of the genes expressed between the hPDLSCs-CTR and hPDLSCs-MOR groups and their fold change. In addition, Figure 4 shows the distribution of the up- and down-regulated genes. The cellular localization of the genes is illustrated in Figure 5 while the Figure 6 shows the interaction of the genes in PINK1-mediated pathway.

### 2.4. Moringin Treatment Modulated the Expression of SOD1, CASP1, Bax, Bcl2 and LC3A/B 

**Figure 7:**
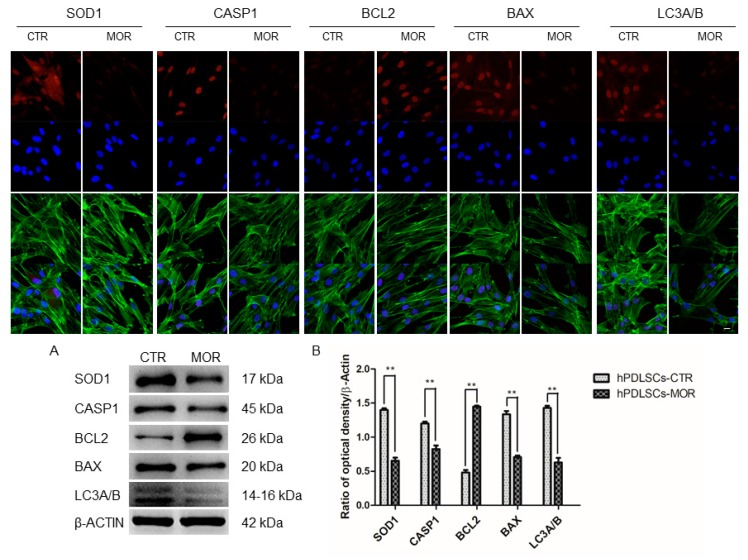
Immunofluorescence analysis in which is shown the expression of SOD1, CASP1, Bax, Bcl2 and LC3A/B in hPDLSCs-CTR and hPDLSCs-MOR. Red fluorescence: specific marker. Green fluorescence: cytoskeleton actin. Blue fluorescence: cell nuclei. Mag: 63X. Scale bar: 10 µm. Western blot analysis confirmed the immunofluorescence results. Protein specific bands of all studied samples (**A**). Graph bars showed the densitometric analysis (**B**). ** *p* < 0.01.

In order to confirm the changes in the expression of the proteins, the immunofluorescence observations were performed under confocal laser scanning microscope. They showed a downregulation of SOD1, CASP1, Bax and LC3 in hPDLSCs-MOR when compared to the hPDLSCs-CTR. On the other hand, Bcl2 is upregulated in hPDLSCs-MOR compared to hPDLSCs-CTR and it shows an opposite behavior (Figure 7). To validate the expression of SOD1, Caspase 1, BCL2, BAX, and LC3A/B proteins the western blotting analysis was performed. In Figure 7, the expression of Caspase 1, SOD1, BAX and LC3A/B were decreased in MOR treated hPDLSCs, while in control sample, an upregulation of BCL2 was evident. 

### 2.5. Mitochondrial Membrane Potential Evaluation

**Figure 8:**
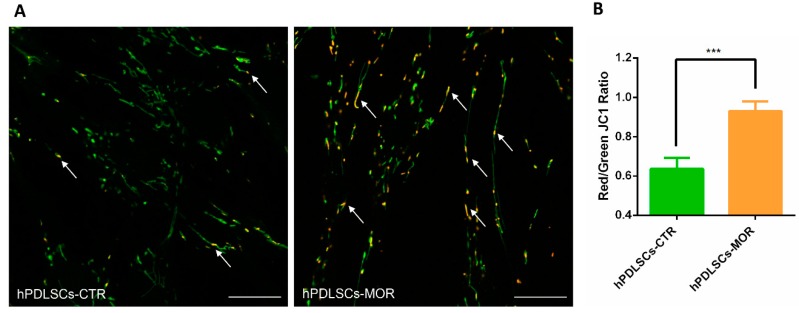
Mitochondrial membrane potential. (**A**) Representative images of hPDLSCs-CTR and hPDLSCs-MOR treated cells stained with JC1. JC-1 fluorescence highlights an increase in polarized mitochondrial membrane potential in hPDLSCs-MOR. Regions with high mitochondrial polarization, from red to yellow fluorescence, indicative of concentration-dependent of J-aggregates are indicated with white arrows. (**B**) Graph of quantitative analysis of Red/Green JC1 ratio. Data are expressed as mean ± S.E.M (hPDLSCs-CTR *n* = 20, hPDLSCs-MOR *n* = 29, *N* = 2; *** *p* < 0.0001). Statistical analysis was performed by unpaired t test with Welch’s correction. Bar = 20 µm.

In order to determine the involvement of mitochondrial membrane potential (ΔΨm) in moringin treatment, hPDLSCs were loaded with the mitochondrial membrane potential probe JC1. The greater ΔΨm, the greater concentration of JC-1 aggregate forms after the mitochondrial uptake which have a red fluorescent emission signal, as opposed to the JC-1 monomer that in condition of low ΔΨm fluoresces green. Figure 8A shows a typical image recorded in which JC1 staining depicted mitochondria structures in hPDLSCs. Nor in control neither in moringin-treated cells we observe a homogenous red or green JC-1 signal from the mitochondria. Instead, the mitochondria were green and red, indicating distinct polarized areas. The proportions of green- and red-emitting mitochondria were not evenly distributed between hPDLSCs-CTR and hPDLSCs-MOR. The red-fluorescing, highly energized mitochondria were proportionally more prevalent in hPDLSCs-MOR (see figure 8A). Quantitative analysis of changes in ΔΨm (Figure 8B), expressed as red/green JC1 fluorescence ratio, put in evidence that in hPDLSCs-MOR the proportion of red fluorescence is increased respect to the hPDLSCs-CTR. (Average values hPDLSCs-MOR and hPDLSCs-CTR: 0.64 ± 0.056 vs 0.93 ± 0.049, S.E.M, respectively).

## 3. Discussion

The progress of stem cell research is clarifying new features of their developmental processes, such as self-renewal, longevity and differentiation capacities *in vitro*. Oral stem cells are widely described in literature for their use in regenerative medicine, for their capacity to undergo multiple processes of differentiation and for their therapeutic potential in repairing damaged tissues [10]. In vitro hPDLSCs showed stable growth and proliferation rate in a culture environment [45,46,47]. Our research group has already conducted several studies on the effects of moringin in hPDLSCs at different concentrations (0.25 µM, 0.5 µM, 1 µM) [48]. Nevertheless, the dose of moringin influences the expression of genes involved in neural differentiation up to 0.5 µM. When the dose is higher (1 µM), the fold change of the genes is not conditioned [8]. Based on this consideration, the dose used in this analysis is 0.5 µM. In this transcriptional study on hPDLSCs, we focused on the effects on moringin in mitophagic and apoptotic processes. Indeed, in physiological condition, mitophagy is a check quality system that preserves healthy mitochondria and eliminates impaired ones [49]. In detail, the correct mitochondrial biogenesis and its maintenance are possible by two processes: fusion and fission. The correct balance between fusion and fission appears to be crucial in the biogenesis of the mitochondria and in their maintenance. Fusion can be stimulated by accumulation of DNA modifications as a consequence of oxidative stress and ROS production. *MFN1* gene is involved in this process and it is downregulated in hPDLSCs-MOR. In particular, it encodes for the protein mitofusin-1 that, during the fusion, changes the morphology of the mitochondria [50]. Fission generates novel mitochondria and promotes the quality control necessary for the cell growth. The analysis of our transcriptome shows that *DNM1L*, involved in fission, is upregulated in hPDLSCs-MOR. It encodes for the dynamin-1-like protein, a GTPase indispensable for the process [51]. Arduino et al. describes that the downregulation of *DNM1L* along with the upregulation of *MFN1* hinds the sharing of mitochondria in dendritic spines [52]. The best characterized pathway of mitophagy is the PINK1-mediated. In healthy conditions, this pathway preserves the mitochondrial homeostasis and does not promote mitophagy (Figure 6A). The gene *PINK1* plays a crucial role in mitophagy as stress sensor. It encodes for the protein PTEN-induced kinase 1 and it is downregulated in hPDLSCs-MOR. Physiologically, PINK1 is firstly translocated into the OMM and later in the inner mitochondrial membrane. Next, PINK1 is cleaved by the Mitochondrial Processing Peptidase (MPP) enzyme [53] and it is finally degraded in the cytosol by the Ubiquitin-Proteasome System [54]. In hPDLSCs-MOR, *PMPCB*, the subunit b of MPP [55], is downregulated. The downregulation of *PMPCB* correlates with the lower expression of PINK1. The MPP recognizes several mitochondrial precursor proteins among which PINK1. In particular, the MPP cleaves the N-terminal of PINK1 thus the C-terminal can be released into the cytosol and finally degraded. Nevertheless, PINK1 can be held in the OMM when the mitochondrial membrane is depolarized due to environmental and stress factors. Consequently, the activity of MPP is obstructed and unhealthy mitochondrial condition is promoted (as illustrated in Figure 6B) [56]. Our results show 21 deregulated genes involved in the pathway of mitophagy among which 15 are downregulated (*PINK1*, *TOMM7*, *SQSTM1*, *NBR1*, *TAX1BP1*, *MFN1*, *OPTN*, *UBB BCL2L13*, *BNIP3L MAP1LC3B*, *GABARAP*, *GABARAPL1*, *GABARAPL2*, *FKBP8*) and 6 are upregulated (*TOMM5*, *CALCOCO2*, *DNM1L*, *UBC*, *UBA52*, *RPS27A*). *PINK1* plays the pivotal role because its accumulation is the trigger of many neurodegenerative diseases, like Parkinson’s [56,57]. In hPDLSCs-MOR are expressed two subunits of the TOM complex, *TOMM7* is downregulated and *TOMM5* is upregulated. Actually, the complex regulates the entrance of the nuclear encoded proteins into the mitochondria thus it achieves a very important activity [58]. In detail, *TOMM7* holds PINK1 to the mitochondrial surface [59], while *TOMM5* seems do not have a specific role in the TOM complex [60]. Furthermore, we also evaluated the mitochondrial membrane potential both in hPDLSCs-CTR and hPDLSCs-MOR. The polarization of mitochondrial surface can be studied by fluorescence analysis [61]. As shown in Figure 8, the JC-1 fluorescence highlights in hPDLSCs-MOR an improvement in the mitochondria membrane polarization.

Interestingly, our transcriptome expresses the *UBB*, *UBC*, *UBA52* and *RPS27A* genes that encode for all the ubiquitins. In particular, the hPDLSCs-MOR shows the upregulation of *UBC*, *UBA52*, *RPS27A* and the downregulation of *UBB*. The ubiquitins play a very important role in molecular signalling. They target toxic and misfolded proteins and allow their degradation through the ubiquitin-proteasome system [62]. Moreover, the ubiquination process contributes to stem cells fate and the upregulation of the ubiquitins may also be correlated with the effect of moringin in stem cells differentiation [63]. When the ubiquitins are recruited in OMM, they are phosphorylated by PINK1 [64]. Consequently, the autophagy receptors are assembled to get started the mitochondrial digestion [65]. In hPDLSCs-MOR are expressed five autophagy receptors (*SQSTM1*, *NBR1*, *OPTN*, *TAX1BP1, CALCOCO2)* among which *SQSTM1*, *NBR1*, *OPTN*, *TAX1BP1* are downregulated while *CALCOCO2* is upregulated. Autophagy receptors are involved in oxidative stress and DNA damage response pathway [66]. Furthermore, they are active in axon homeostasis and promote the replacement of the inflammatory proteins [67]. The protein structure of the autophagy receptors is characterized by an ubiquitin domain that identifies the phosphorylated ubiquitins and by a LC3-interacting region (LIR) motif [68]. The LIR motif also characterizes the structure of receptors that take part in OMM receptor-mediated pathway. The OMM receptors can promote the mitophagy without mediation of ubiquitin or autophagy receptors [69]. The hPDLSCs-MOR show a downregulation of *BNIP3L*, *BCL2L13* and *FKBP8* genes that encode for OMM receptors [70]. Finally, the LIR motifs of autophagy and OMM receptors binds the phagophore, the precursor of the autophagosome [71]. The proteins of the LC3/GABARAP family entail the biogenesis and maturation of the autophagosome [72]. Interestingly, hPDLSCs-MOR show a downregulation of *MAP1LC3B* (LC3B), *GABARAP*, *GABARAPL1* and *GABARAPL2* genes which encode for proteins of LC3/GABARAP family. Thus, the downregulation of these genes may obstruct the phagophore formation. Furthermore, the expression of LC3A/B was evaluated by immunofluorescence that confirms the downregulation (Figure 7).

In addition, the overproduction of ROS and the oxidative stress can promote mitophagy [73] and lead to apoptosis [74]. In hPDLSCs-MOR six genes that encode for pro-apoptotic proteins are downregulated (*CASP1*, *CASP4*, *CASP6*, *CASP7*, *CASP8, BAX*) and one is upregulated (*CASP2*). In particular, CASP1 and CASP4 mediate the activation of inflammosome. *CASP2* and *CASP8* are involved in the initiatory effects of apoptosis while *CASP6* and *CASP7* are the effector caspases. Moreover, *BAX* is a pro-apoptotic gene that handles the permeabilization of the OMM and it is downregulated. The overall downregulation of pro-apoptotic genes reduces of the apoptotic process [75]. Furthermore, the immunofluorescence analysis performed for CASP1 and BAX confirms their downregulation. In hPDLSCs-MOR the anti-apoptotic genes *BCL2L12* and *MCL1* are upregulated. They encode for pro-survival proteins of the Bcl-2 family that bind the pro-apoptotic proteins. This interaction prevents the release of cytochrome C in the cytosol and consequently the apoptosis [76,77]. BCL2 were analyzed with immunofluorescence that demonstrates its upregulation. The cytochrome C is encoded by *CYCS* gene that is upregulated in hPDLSCs-MOR. In addition, in hPDLSCs-MOR *SOD1* is downregulated. It encodes for a protein of the superoxide dismutase family and its downregulation correlates with the reduction of the oxidative stress [78]. The immunofluorescence analysis also shows a downregulation for SOD1. Our group has already verified the inhibition of the apoptosis in hPDLSCs treated with moringin at the same concentration (0.5 µM) [48]. 

Finally, 22 genes were investigated. The hPDLSCs-MOR express 10 genes that are not expressed in hPDLSCs-CTR (*HIGD1C*, *PAGE2*, *SNORA1*, *SNORA32*, *SNORD104*, *SNORD114-22*, *SNORA116-15*, *SNORD116-23, TEX29*, *C21orf140*) while 12 genes are expressed in hPDLSCs-CTR and are absent in hPDLSCs-MOR (*MT1A*, *SPC25*, *OIP5*, *CDCA3*, *GINS2*, *PKMYT1*, *ZBTB16*, *HIST1H3F*, *HIST2H3A*, *HIST2H3C*, *HIST2H4B, C1orf53*). In particular, *HIGD1C* is an important paralog of the gene *HIGD1A* that is upregulated in hPDLSCs-MOR. *HIGD1A* inhibits the release of cytochrome C that reduces the activation of caspases and it finally results into survival effects [79]. *PAGE2* gene encodes for a P antigen protein that was studied as possible target in psychotic episodes [80]. *SNORA1*, *SNORA32*, *SNORD104*, *SNORD114-22*, *SNORA116-15*, *SNORD116-23* are small nucleolar RNAs (snoRNAs) that act as regulatory factors by methylation and pseudouridylation [81]. *MT1A* gene encodes for a metallothionein protein that is usually involved in metal toxicity and oxidative stress [82]. *SPC25*, *OIP5*, *CDCA3, GINS2, PKMYT1, ZBTB16* genes are involved in cell cycle progression and DNA transcription. *HIST1H3F*, *HIST2H3A*, *HIST2H3C*, *HIST2H4B* genes encode for histones. Finally, *TEX29*, *C21orf140, C1orf53* genes are open reading frame. Very little literature is present about them but they do not seem related to neurological disorders.

## 4. Materials and Methods 

### 4.1. Purification of Moringin

The extraction of moringin by *M. Oleifera* (fam. *Moringaceae*) seeds (cake powder PKM2 provided by Indena India Pvt. Ltd.; Bangalore, India) was performed at the Bologna laboratory (CREA-AA; previously CIN) through established methods and the molecular structure was proved by nuclear magnetic resonance (NMR) spectroscopic analyses [83,84].

### 4.2. Ethic Statement

The study was performed in accordance with guidelines of the Helsinki declaration (2013). The protocol used for cell isolation and culture, was approved by the Ethical Committee at the Medical School, “G. d’Annunzio” University, Chieti, Italy (number 266/April 17, 2014). Individuals recruited in the study signed the informative consent form prior tissue collection and all experiments were accomplished according to relevant guidelines and regulations. 

### 4.3. Cell Culture and Moringin Pretreatment

Cells of periodontal tissue were collected scraping a third coronal root surface using Gracey’s curette [85]. The three individuals enrolled in the study were healthy and in the absence of oral and systemic diseases. After collection, hPDLSCs were cultured using MSCGM-CD medium (mesenchymal stem cell growth medium chemically defined) (Lonza, Basel, Switzerland) and were maintained in an incubator at 37 °C in a humidified atmosphere of 5% CO_2_ in air. The hPDLSCs were observed at the light microscopy DMIL and DM 2000 (Leica Microsytem, Milan, Italy) [86]. The cells were treated after reaching the 80% of confluence in a Petri dish. The cells at passage 2 were seeded for all experiments with a density of 300,000 cells/cm^2^. In order to accomplish transcriptomic analysis, hPDLSCs were treated with 0.5 µM of moringin (dissolved in 0.1% DMSO) for 48 h.

Morphological evaluation of treated and untreated hPDLSCs was performed using SEM. Samples were processed as previously reported by Gugliandolo et al. [87]. Finally, cells were mounted on alluminium stubs and gold-coated in an Emitech K550 (Emitech Ltd. Ashford, UK) sputter-coater before imaging by means Zeiss SEM EVO 50 (Zeiss, Jena, Germany). Cell were processed for immunofluorescence observations as following described. Samples were fixed with a solution of 4% paraformaldehyde diluted in 0.1M sodium phosphate buffer (PBS, Lonza). To highlight the cytoskeleton actin samples were incubated with the Alexa Fluor 488 phalloidin green fluorescence conjugate (1:400, Molecular Probes, Eugene, Oregon, USA) for 1 h. After washing cells were incubated with TOPRO (1:200, Molecular Probes) for 1 h at 37 °C [47] to mark cell nuclei. Samples were observed under Zeiss LSM800 confocal system (Zeiss, Jena, Germany) [88].

The viability rate of treated and untreated hPDLSCs were determined by means of the 3-(4,5-dimethylthiazolyl-2)-2,5-diphenyltetrazoliumbromide (MTT) method, at different time point (24, 48 and 72 h), as previously described [89]. Cell proliferation rate was also evaluated by the doubling time of Trypan blue harvested cells at 24, 48 and 72 h of culture and was calculated by using a software available online (http://www.doubling-time.com) [90]. All the experiments were performed in triplicate.

### 4.4. Statistical Analysis

All data were visualized by barplots. The statistical analysis was performed using ANOVA test with GraphPad Prism 6.0 software (GraphPad Software, La Jolla, CA, USA). The p-value threshold used to validate the hypothesis was p < 0.05.

### 4.5. Total RNA Extraction and cDNA Library Preparation 

The Reliaprep RNA cell Miniprep System (Promega, Madison, WI, USA) was utilized for the extraction of the total RNA from all samples and each of them was treated with 0.1% of DMSO. In accordance with the TruSeq RNA Access library kit protocol (Illumina, San Diego, CA, USA), 40 ng of total RNA has been fragmented by using a thermal cycler to 94 °C for 8 min. The fragments obtained (>200 nt) have been used for the synthesis of the first strand cDNA by SuperScript II reverse transcriptase (Invitrogen, Carlsbad, CA, USA). Next, by using the Second Strand Marking Master Mix, a double strand cDNA has been synthesized by incubation at 16°C for 1 h and then purified by AMPure XP beads to remove the reaction mix. Fragments adenylation at the 3’ ends was performed to permit the ligation of the complementary adapters in order to avoid chimera generation. The samples identification and them preparation for flow cell hybridization was performed through the ligation of Adapter-Indexes to double strand cDNA fragments. After the purification by a clean-up step, a first PCR amplification was accomplished in accordance with the following program: denaturation at 98 °C for 30 s, 15 cycles composed of 98 °C for 10 s, 60 °C for 30 s, 72 °C for 30 s and extension at 72 °C for 5 min. To select and enrich specific regions of interest, a step of hybridization reaction was performed to mix exome capture probes with cDNA library. Later, in order to obtain a pool of different indexing libraries, by using 200 ng of each one, a hybridization reaction was accomplished in accordance with the following program: 95 °C for 10 min, an incubation consisting of 18 cycles of 1 min, starting at 94 °C and decreasing 2 °C per cycle and a final step of 58 °C for 90 min. For the sample pool purification streptavidin conjugated magnetic beads were utilized and, a second hybridization reaction, followed by another streptavidin purification, has been accomplished prior the final PCR amplification performed with the following program: 98 °C for 30 s, 10 cycles: 98 °C for 10 s, 60 °C for 30 s, and 72 °C for 30 s, and 72°C for 5 min. A last clean-up was performed in order to obtain the final cDNA library purified. Lastly, the cDNA library has been qualitatively validated by Bioanalyzer instrument (Agilent High Sensitivity DNA kit, Richardson, TX, USA) and quantitatively by Real-Time PCR KAPA Library Quantification Kit-Illumina/ABI Prism® (Kapa Biosystems, Inc., Wilmington, MA, USA). After, the library has been denatured by 2 N NaOH and diluted in order to reach a final concentration of 12 pM. MiSeq Reagent Kit v3 has been used for sequencing on the Illumina MiSeq Instrument, by setting a single read. 

### 4.6. Data Processing

The CASAVA software was used to produce the “FASTQ” format file. The alignment of the reads was achieved by means of “STAR” tool against the “homo sapiens UCSC hg19” reference genome. Cufflinks Assembly & DE package version 2.0.0 was used to perform the statistical evaluation of the genes obtained by the alignment. The normalization of the samples was calculated using the FPKM (fragment per kilobase of exon per million fragmented mapped) strategies: (1000 x read count)/(number of gene covered bases x number of mapped fragments in million).

### 4.7. Immunofluorescence Analysis

The hPDLSCs-CTR and hPDLSCs-MOR were processed as previously reported by Trubiani et al. (2012) [88]. Primary monoclonal antibodies anti-human Caspase 1 (1:200, rabbit) (Abcam, Milan, Italy), anti-human SOD1 (1:200, rabbit) (Abcam), anti-human Bax (1:100, rabbit) (Cell Signaling Technology, Milan, Italy), anti-human Bcl2 (1:200, rabbit) (Cell Signaling Technollogy, Milan, Italy), and anti-human LC3A/B (1:100, rabbit) (Cell Signaling Technology) were used, followed by Alexa Fluor 568 conjugated goat anti rabbit as secondary antibodies (1:200; ThermoFisher, Life Tech., Monza, MB, Italy) for 1 hr at 37 °C. Subsequently cells were incubated with AlexaFluor 488 phalloidin green fluorescence conjugate (1:200; ThermoFisher, Life Tech.), to evidence cytoskeleton actin. Cell nuclei were stained with TOPRO (1:200; ThermoFisher) for 1 hr at 37 °C. Glass coverslips were placed face down on glass slides and mounted with Prolong antifade (ThermoFisher, Life Tech.) [91]. Samples were observed by means of a Zeiss LSM800 confocal system, connected to an inverted Zeiss Axiovert 200 microscope equipped with a Plan Neofluar oil-immersion objective. Images were collected using an argon laser beam with excitation lines at 488 nm and a helium-neon source at 543 and 633 nm. Post-acquisition image analyses were carried out with a Zeiss ZEN software. 

### 4.8. Western Blot Analysis

Proteins (60 μg) from all sample groups were processed as previously described [92]. Proteins were separated on SDS-PAGE and subsequently transferred to nitrocellulose sheets using a semidry blotting apparatus. Sheets were saturated for 60 min at 37 °C in blocking buffer (1xTBS, 5% milk, 0.05% Tween-20), then incubated overnight at 4 °C in blocking buffer containing primary antibodies to SOD1 (1:100, Abcam), Caspase 1 (1:100, abcam), BCL2 (1:100, Santa Cruz Biotechnology, Dallas, Texas, USA), BAX (1:100, Cell Signaling Technology), LC3A/B (1:100, Cell Signaling Technology) and β-actin (1:1000, Santa Cruz Biotechnology). After four washes in TBS containing 0.1% Tween-20, samples were incubated for 30 min at room temperature with peroxidase-conjugated secondary antibody diluted 1:1000 in 1× TBS, 5% milk, 0.05% Tween-20. Bands were visualized by the ECL method. The level of recovered protein was measured using the Bio-Rad Protein Assay (Bio-Rad Laboratories, Hercules, CA, USA) according to the manufacturer’s instructions.

### 4.9. Measurement of Mitochondrial Membrane Potential

We evaluated ΔΨm using 5,5′,6,6′-tetrachloro-1,1’3,3’-tetraethylbenzamidazol-carboncyanine (JC-1, Invitrogen). JC-1 accumulates in polarized mitochondria, and when exited at 488 nm shows a double emission in the green (530 nm emission) fluorescent monomers at low membrane potentials and as orange/red (590 nm emission) fluorescent aggregates at high membrane potentials [93]. For JC-1 staining 104 hPDLSCs were seeded in 35 mm imaging dish (µ-Dish, ibidi GmbH, Gräfelfing, Germany) and incubated in culture media with 5 µM JC-1 for 5 min at 37 °C in a cell culture incubator. At the end of incubation time the cells were washed twice and observed with Normal External Solution (NES) containing (in mM): 125 NaCl, 5 KCl, 1 MgSO_4_, 1 KH_2_PO_4_, 5.5 glucose, 1 CaCl_2_, 20 HEPES, pH 7.4. Confocal images were randomly acquired using a Zeiss LSM800 microscope (Carl Zeiss), equipped with an Axio-obserber.D1 inverted microscope and an objective W-Plan-Apo 63X/1.4 DIC. The red/green fluorescence intensity ratios, were independently calculated for treated and control hPDLSCs using Fiji distribution of ImageJ [94].

## 5. Conclusions

The treatment of hPDLSCs with moringin downregulates most of the genes involved in mitophagy. Noteworthily, moringin downregulates both the trigger of the process (*PINK1*) and the genes that regulate the formation of phagophore (*MAP1LC3B*, *GABARAP*, *GABARAPL1*, *GABARAPL2*). Moreover, the genes involved in oxidative stress are not expressed except for *SOD1* that is also downregulated. The immunofluorescence analysis supports the reduction of the expression of SOD1, CASP1, BAX and LC3A/B and the increase of the anti-apoptotic protein BCL2. In addition, our results confirm that moringin pretreatment is not cytotoxic. Consequently, its beneficial effects seem to improve hPDLSCs for possible uses in stem cell therapies. In particular, it could benefit more the disorders that have the oxidative stress as etiophatogenesis mechanisms, such as the neurodegenerative diseases. 

## Figures and Tables

**Table 1:** Genes expressed both in hPDLSCs-CTR and hPDLSCs-MOR involved in the mitophagy pathway.

Genes	hPDLSCs-CTR	hPDLSCs-MOR	Fold Change	Q-Value	Protein	Source
PINK1	37.36	25.27	−0.56	<0.01	Serine/threonine-protein kinase PINK1	Reactome
PMPCB	10.45	8.24	−0.34	0.01	Mitochondrial-processing peptidase subunit beta	[29,30]
TOMM7	84.53	70.94	−0.25	0.04	Mitochondrial import receptor subunit TOM7 homolog	Reactome
TOMM5	11.09	19.32	0.80	0.01	Mitochondrial import receptor subunit TOM5 homolog	Reactome
SQSTM1	197.31	102.56	−0.94	<0.01	Sequestosome-1	Reactome
CALCOCO2	36.71	48.12	0.39	<0.01	Calcium-binding and coiled-coil domain-containing protein 2	[29,30,31,32,33,34]
NBR1	42.99	34.24	−0.33	<0.01	Next to BRCA gene 1 protein	[29,30,31,33,35,36,37]
TAX1BP1	35.90	27.99	−0.36	<0.01	Tax1-binding protein 1	[29,30,31,33,34]
MFN1	10.97	8.36	−0.39	0.01	Mitofusin-1	Reactome
DNM1L	12.24	18.06	0.56	<0.01	Dynamin-1-like protein	[30,31,33,34,36]
OPTN	88.81	67.17	−0.40	<0.01	Optineurin	[30,31,32,33,34,35,36]
UBB	1415.11	986.86	−0.52	<0.01	Polyubiquitin-B	Reactome
UBC	554.15	681.83	0.30	<0.01	Polyubiquitin-C	Reactome
UBA52	237.50	261.25	0.14	<0.01	Ubiquituin-60S ribosomial protein L40	Reactome
RPS27A	837.70	933.41	0.16	<0.01	Ubiquitin-40S ribosomial protein S27a	Reactome
BCL2L13	11.03	8.83	−0.32	0.01	Bcl-2-like protein 13	[30,31,33,34]
BNIP3L	15.53	11.81	−0.40	<0.01	BCL2/adenovirus E1B 19 kDa protein-interacting protein 3-like	[30,31,33,34,36,37]
MAP1LC3B	26.95	16.28	−0.73	<0.01	Microtubule-associated proteins 1A/1B light chain 3B	Reactome
GABARAP	252.95	175.99	−0.52	<0.01	γ-Aminobutyric acid receptor-associated protein	[29,30,31,33,34]
GABARAPL1	18.22	11.21	−0.70	<0.01	γ-Aminobutyric acid receptor-associated protein-like 1	[29]
GABARAPL2	50.42	38.38	−0.39	0.01	γ-Aminobutyric acid receptor-associated protein-like 2	[29]
FKBP8	84.95	69.58	−0.29	<0.01	Peptidyl–prolyl *cis-trans* isomerase FKBP8	[33]

For each gene in the column *Genes*, the column *hPDLSCs-CTR* highlights the level of expression of the transcript in the not treated hPDLSCs while in the column *hPDLSCs-MOR* the level after the treatment. The *Fold change* is obtained by Log_2_ (*hPDLSCs-MOR*/*hPDLSCs-CTR*). The *Q-value* was used to choose the level of significance (<0.05). The *Protein* column shows the name of the encoded protein by UniProt. The last column highlights the source from which the gene was chosen. All the values are rounded to the second decimal digit.

**Table 2:** Genes expressed both in hPDLSCs-CTR and hPDLSCs-MOR involved in apoptotic pathway.

Genes	hPDLSCs-CTR	hPDLSCs-MOR	Fold Change	Q-Value	Protein	Source
BCL2L12	1.42	3.63	1.35	<0.01	Bcl-2-like protein 12	[38,39,40,41]
BAX	62.22	39.37	−0.66	<0.01	Apoptosis regulator BAX	[38,39,40,41,42,43]
MCL1	13.39	17.22	0.36	<0.01	Induced myeloid leukemia cell differentiation protein Mcl-1	[38,39,40,41]
CYCS	3.82	7.64	1.00	<0.01	Cytochrome c	[38,39,40,41,42,43,44]
CASP1	17.76	6.60	−1.43	<0.01	Caspase-1	[38]
CASP2	5.72	9.34	0.71	<0.01	Caspase-2	[44]
CASP4	104.74	92.25	−0.18	0.01	Caspase-4	[44]
CASP6	4.66	1.85	−1.34	0.01	Caspase-6	[39]
CASP7	9.81	7.15	−0.46	0.01	Caspase-7	[38]
CASP8	12.86	8.18	−0.65	<0.01	Caspase-8	[38]
SOD1	470.72	404.41	−0.22	<0.01	Superoxide dismutase [Cu-Zn]	[44]

For each gene in the column *Genes*, the column *hPDLSCs-CTR* highlights the level of expression of the transcript in the not treated hPDLSCs while in the column *hPDLSCs-MOR* the level after the treatment. The *Fold change* is obtained by Log_2_ (*hPDLSCs-MOR*/*hPDLSCs-CTR)*. The *Q-value* was used to choose the level of significance (<0.05). The *Protein* column shows the name of the encoded protein by UniProt. The last column highlights the source from which the gene was chosen. All the values are rounded to the second decimal digit.

## Supplementary Materials

The following are available online, Table S1: Full list of differential expressed genes between hPDLSCs-CTR and hPDLSCs-MOR.
